# Alzheimer’s Disease Susceptibility Genes *APOE* and *TOMM40*, and Hippocampal Volumes in the Lothian Birth Cohort 1936 

**DOI:** 10.1371/journal.pone.0080513

**Published:** 2013-11-15

**Authors:** Donald M. Lyall, Natalie A. Royle, Sarah E. Harris, Mark E. Bastin, Susana Muñoz Maniega, Catherine Murray, Michael W. Lutz, Ann M. Saunders, Allen D. Roses, Maria C. del Valdés Hernández, John M. Starr, David. J. Porteous, Joanna M. Wardlaw, Ian J. Deary

**Affiliations:** 1 Centre for Cognitive Ageing and Cognitive Epidemiology, University of Edinburgh, Edinburgh, United Kingdom; 2 Brain Research Imaging Centre, Division of Neuroimaging Sciences, University of Edinburgh, Edinburgh, United Kingdom; 3 Department of Psychology, University of Edinburgh, Edinburgh, United Kingdom; 4 Medical Genetics Section, Centre for Genomics and Experimental Medicine and MRC Institute of Genetics and Molecular Medicine, University of Edinburgh, Edinburgh, United Kingdom; 5 Scottish Imaging Network, A Platform for Scientific Excellence (SINAPSE) Collaboration, Division of Neuroimaging Sciences, University of Edinburgh, Edinburgh, United Kingdom; 6 Joseph & Kathleen Bryan Alzheimer’s Disease Research Center, Department of Neurology, Duke University Medical Center, Durham, North Carolina, United States of America; 7 Zinfandel Pharmaceuticals, Inc., Durham, North Carolina, United States of America; 8 Alzheimer Scotland Dementia Research Centre, University of Edinburgh, Edinburgh, United Kingdom; Cambridge Institute for Medical Research, United Kingdom

## Abstract

The *APOE* ε and *TOMM40* rs10524523 (‘523’) variable length poly-T repeat gene loci have been significantly and independently associated with Alzheimer’s disease (AD) related phenotypes such as age of clinical onset. Hippocampal atrophy has been significantly associated with memory impairment, a characteristic of AD. The current study aimed to test for independent effects of *APOE* ε and *TOMM40* ‘523’ genotypes on hippocampal volumes as assessed by brain structural MRI in a relatively large sample of community-dwelling older adults. As part of a longitudinal study of cognitive ageing, participants in the Lothian Birth Cohort 1936 underwent genotyping for *APOE* ε2/ε3/ε4 status and *TOMM40* ‘523’ poly-T repeat length, and detailed structural brain MRI at a mean age of 72.7 years (standard deviation = 0.7, N range = 624 to 636). No significant effects of *APOE* ε or *TOMM40* 523 genotype were found on hippocampal volumes when analysed raw, or when adjusted for either intracranial or total brain tissue volumes. In summary, in a large community-dwelling sample of older adults, we found no effects of *APOE* ε or *TOMM40* 523 genotypes on hippocampal volumes. This is discrepant with some previous reports of significant association between *APOE* and left/right hippocampal volumes, and instead echoes other reports that found no association. Previous significant findings may partly reflect type 1 error. Future studies should carefully consider: 1) their specific techniques in adjusting for brain size; 2) assessing more detailed sub-divisions of the hippocampal formation; and 3) testing whether significant *APOE*-hippocampal associations are independent of generalised brain atrophy.

## Introduction

Dementia is a growing world-wide problem, and it is important to understand the risk factors and mechanisms underlying the disease. The major sub-type of dementia is Alzheimer’s disease (AD). A key brain region involved in the illness is the hippocampus, and hippocampal volumetric atrophy has been used as an indicator of AD risk [1,2]. Two genetic risk factors are in the *APOE* and *TOMM40* genes [3]. The present study concerns the association between specific variants in these genes and hippocampal volume in non-demented older people, in an attempt to investigate further possible links between these candidate genes and a prominent brain phenotype thought to be indicative of AD risk. We first introduce these genes and then the hippocampus and its part in dementia, including genetic risk factors.

### Apolipoprotein-e (*APOE*)

The *APOE* ε haplotype (commonly and hereinafter referred to as ε ‘genotype’) is composed of two single nucleotide polymorphisms (SNPs); rs429358, which causes a Cys130Arg substitution; and rs7412, which causes an Arg176Cys substitution [4]. Different combinations of the rs429358/rs7412 SNPs form the ε2 (Cys/Cys respectively), ε3 (Cys/Arg) and ε4 (Arg/Arg) haplotypes [5]. Of these, the ε3 allele is the most common (frequency ~78.3%), followed by ε 4 (~14.5%) and ε2 (~6.4%) [6]. 

*APOE* plays a role in the transport and metabolism of lipid in the brain [7][8]. The ε4 allele is the deleterious ‘risk’ variant for cognitive ageing [9] and diagnosis of AD [10] compared with ε3 (‘neutral’), and ε2 (putatively ‘protective’).

The *APOE* ε4 allele may contribute to phenotypic differences through different and/or interactive mechanisms, by: i) moderating processes that lead to an accumulation of 42-residue amyloid-beta (‘Aβ’) plaques in the brain [11,12]; ii) disrupting the normal transport and catabolism of cholesterol, required for the development and maintenance of neurons and myelin [5,11]; and iii) via second order associations with vascular diseases that affect the brain, such as atherosclerosis [13,14]. Independent genetic factors may play an interactive or moderating role in terms of the association between *APOE* ε genotype and clinical AD/cognitive decline. 

### Translocase of Outer Mitochondrial Membrane 40 (*TOMM40*)

The *TOMM40* gene is located adjacent to *APOE* [15] and several SNPs in the two genes are in significant linkage disequilibrium (LD); e.g. rs429358 and 36 SNPs within ±1.17 KB of the *APOE* region including 15 *TOMM40* SNPs; average D’ = 0.91, r^2^ = 0.22, n = 1262 [16]. 

*TOMM40* encodes the channel-forming subunit of the translocase of outer mitochondrial membrane (TOM) complex [15,17]. This complex imports precursor proteins into mitochondria [18], and dysfunction of this biological pathway may play a role in cognitive decline and AD pathology (“mitochondrial cascade hypothesis” [19]). Apoe and Tomm40 proteins may interact to affect mitochondrial dynamics although the precise mechanisms underlying this are unclear [3,20]. 

The rs10524523 locus (hereinafter ‘*523*’) in *TOMM40* is characterised by a variable number of T residues (poly-T repeats) which can be grouped into ‘Short’ (<20; ‘S’), ‘Long’ (20-29; ‘L’), and ‘Very-Long’ (≥30; ‘VL’) [21]. Using phylogenetic mapping analyses, Roses et al.[3] showed that poly-T repeat length was strongly linked with the *APOE* ε genotype; ε4 is linked to L, with ε3 linked to either S or VL alleles. The rarer ε2 allele appeared to show similar linkage to S or VL alleles in *TOMM40* 523, as per ε3, although further research is required. 

The functional significance of *TOMM40* 523 poly-T repeat length is the subject of ongoing research. Bekris et al.[20] (N = 32) reported that specific *TOMM40* 523 variants were associated with lower *TOMM40*, but not *APOE*, gene expression in SHSY5Y neuronal cell lines, indicating a role for this locus in *TOMM40* promoter silencer/enhancer activity. Studies have reported association between the *TOMM40 523* repeat and brain-related phenotypes such as risk of AD diagnosis [22], earlier/later age of AD clinical onset [3,21,22], cortisol levels [23] and cognitive change in older age [24], independent of *APOE*.

### The hippocampus

The hippocampal formation of the brain includes the dentate gyrus, subiculum, entorhinal cortex, cornu ammonis (CA) areas 1-4 and the hippocampus proper [25,26]. The hippocampus itself occupies the floor of the temporal horn of the lateral ventricle. It is typically 4.0 to 4.5 cm long and around 1.5 cm wide [27].

Braak and Braak [28] outline six stages of neurofibrillary change in AD pathophysiology, based on the observed accumulations of neurofibrillary tangles, neuropil threads and amyloid beta plaques. AD-like neurofibrillary changes have been reported in the human brain in the absence of significant cognitive decline [29,30], and are apparent first in the hippocampal formation [25,31]. Hippocampal atrophy is associated with a common symptom of AD, namely memory impairment [1,2].

Hippocampal volumes and *APOE* ε genotype in healthy older adults has been examined by a number of small (N range = 20 to 134) brain MRI studies, which vary between showing significant and null effects ^e.g^[32–36]. and a few much larger reports including several hundred participants, which also show varying results [37–42]. These larger reports are likely to be more reliable, and are summarised in Table 1. Specifically, possession of the *APOE* ε4 allele has occasionally been significantly associated with lower hippocampal volumes in cross-sectional samples of older adults.

**Table 1:** Summary of previous large cross-sectional studies (i.e. N > 135) examining *APOE* ε4 genotype and hippocampal volumes in non-demented, community dwelling older adults.

Authors	Technique used in correcting hippocampal volumes for head size	Sample N	Mean age in years (Standard Deviation)	Covariates	Main findings	Statistics (hippocampal volumes)
LeMaitre et al.[37]	Hippocampal volumes expressed as a percentage fraction of intracranial volume.	750	69.4 (2.9)	Gender (controlled), age, education, diagnosis of hypertension, Mini Mental State Exam score (no group differences; *P* > 0.05)	Significant deleterious effect of ε4/ε4 genotype (vs. non-ε4 carriers).	Left: ε4/ε4 = 0.23%, vs. non-ε4/ = 0.26% (p <0.001). Right ε4/ε4 = 0.22% vs. non-ε4 = 0.24% (p = 0.006)
Den Heijer et al.[38]	Midsagittal area included as model covariate.	949	72.3 (7.0)	Age, gender.	Significant deleterious effect of ε4 allele presence (vs. non-ε4 carriers).	Left: ε4+ difference to ɛ3/ɛ3 genotype = -0.11 millilitres. Right: ε4+ difference to ɛ3/ɛ3 genotype = -0.11 milliliters.
Cherbuin et al.[39]	Intracranial volume included as model covariate.	331	62.6 (1.4)	Age, gender, education.	No significant main effects of ε4 allele (P > 0.05).	-
Panizzon et al.[40]	Intracranial volume included as a model covariate.	375	55.9 (2.6)	Relatedness between twins, handedness, age.	No significant main effects of ε4 allele (P > 0.05).	-
Ferencz et. al.[41]	Intracranial volume included as a model covariate.	424	69.9 (8.6)	Age.	No significant main effects of ε4 allele (P > 0.05).	-
Hostage et al. [42]	Intracranial volume included as a model covariate.	198	76.0 (0.5)	Age	No significant main effects of ε4 allele (P > 0.05).	-

Note. The age mean and standard deviation data provided above for Lemaitre et al., Den Heijer et al. and Hostage et al. are weighted estimates; see those reports for exact age data

*TOMM40* 523 poly-T repeat length is linked to *APOE* genotype, and as far as we are aware, only one study has so far examined the independent effects of the *TOMM40* 523 gene locus on hippocampal volumes in healthy older adults. Johnson et al.[43] tested for an effect of poly-T repeat length genotype on a whole-brain voxel-wise comparison of grey matter volumes in participants with the *APOE* ε3/ε3 genotype (N = 117, mean age = 55.47 years, SD = 6.00), and found no significant effect of poly-T repeat genotype on hippocampal volume. A recent large-scale genome wide association study in healthy adults (N = 5776) reported no significant associations with SNPs in the *TOMM40* gene however did not directly analyse the *TOMM40* 523 poly-T repeat locus [44]. 

The Lothian Birth Cohort 1936 [45] (LBC1936) is large group of relatively healthy older adults of a narrow age range, that have undergone detailed brain MRI and *APOE*/*TOMM40* genotyping. The current study aims to add to the literature by investigating: 1) the effects of *APOE* ε and 2) the independent effects of the *TOMM40 523* poly-T repeat length, on hippocampal volumes in the LBC1936.

## Methods

### Sample and procedure

The LBC1936 consists of 1091 community-dwelling adults most of whom completed the Moray House Test no.12 of verbal reasoning as part of the Scottish Mental Survey of 1947 at a mean age of 11 years [46]. The recruitment and testing of this sample has been described in detail elsewhere [45,47]. In the first wave of the LBC1936 study (‘Wave 1’), around age 70 years, they underwent detailed cognitive, sociodemographic, and physical assessments [45]. The sample was generally healthy; participants with current acute severe illness were excluded. Around three years later, members of the cohort returned for re-testing. At that time detailed structural brain MRI was added in what was the second wave of the study (‘Wave 2’[48]). Participants were screened for cognitive impairment with the Mini-Mental State Examination (MMSE), with scores under 24 used to indicate possible dementia [49]. Diagnoses of clinical disorders were elicited via interview. 

### Ethics statement

Ethics permission for the study protocol was obtained from the Multi-Centre Research Ethics Committee for Scotland (MREC/01/0/56) and from The Lothian Research Ethics Committee (LREC/2003/2/29), in accordance with the Declaration of Helsinki, and all participants gave written, informed consent [45]. 

### Genotyping

DNA was isolated from whole blood, and the *APOE* SNPs rs7412 and rs429358 SNPs genotyped with TaqMan technology. These two SNPs form the *APOE* ε2/ε3/ε4 haplotype. *TOMM40* 523 poly-T repeat length was genotyped by the laboratory of Dr. Ornit Chiba-Falek (Duke University, NC, USA) using a method described previously [50]. Briefly, each genomic DNA sample was amplified by the polymerase chain reaction (PCR) using fluorescently labelled forward 5’FAM-TGCTGACCTCAAGCTGTCCTG-3’ and reverse 5’-GAGGCTGAGAAGGGAGGATT-3’primers. Genotypes were determined on an ABI 3730 DNA Analyzer, using GeneMapper version 4.0 software (Applied Biosystems, Foster City, California, USA) for fragment analysis by the amplified fragment length polymorphism method validated for research studies and commercially available. 

### MRI analysis

LBC1936 MRI data acquisition and processing is described in detail by the protocol paper, by Wardlaw et al. [48]. Participants underwent whole brain structural MRI acquired using a GE Signa Horizon 1.5 T HDxt clinical scanner (General Electric, Milwaukee, USA) equipped with a self-shielding gradient set (33 mT m^-1^ maximum gradient strength) and manufacturer supplied 8-channel phased-array head coil [48]. In addition to standard structural T2-, T2*- and FLAIR-weighted MRI, the imaging protocol included a high-resolution T1-weighted volume sequence acquired in the coronal plane with field-of-view 256 × 256 mm, imaging matrix 192 × 192 (zero-filled to 256 × 256), 160 1.3mm thick slices giving 1 × 1 × 1.3 mm voxel dimensions. The repetition, echo and inversion times were 10, 4 and 500 ms respectively. 

 Left and right hippocampal volumes were semi-automatically segmented using FSL (http://fsl.fmrib.ox.ac.uk) with manual correction. High resolution T1-weighted volume scans were passed through FSL’s FIRST automated segmentation software to create volume masks for left and right hippocampus, based on standard protocols (http://www.fmrib.ox.ac.uk/fsl/first/index.html). Noise reduction was applied to the resulting volumes which were then registered using FSL FLIRT to a locally developed template of similarly older brains. A second registration to an optimised sub-cortical mask was then performed followed by boundary correction, with the resulting masks visually assessed for accuracy and manually edited using Analyse 9.0 (http://www.analyzedirect.com) where discrepancies occurred. This method has been validated against the ‘gold-standard’ approach of manual tracing [48].

Intracranial volume measurements were obtained semi-automatically using the T2*-weighted sequence [48], and included the contents of the inner skull, with an inferior limit in the axial slice just superior to the tip of the odontoid peg at the foramen magnum and superior to the inferior limits of the cerebellar tonsils. Total brain tissue volumes were obtained using T2*-weighted with FLAIR to obtain a surrogate cerebrospinal fluid (CSF) mask (volume) that contained CSF, venous sinuses and the meninges. The volume of ‘CSF+veins+meninges’ was used to estimate total brain tissue volume by subtracting it from intracranial volume [48].

## Statistical Analysis

### Statistical models

All models controlled for gender and age in days at neuroimaging. An online calculator was used to perform tests of Hardy-Weinberg equilibrium and determine minor allele frequencies [51]. Data were otherwise analysed with the Predictive Analytics SoftWare (version 17; http://www-01.ibm.com/software) statistics programme. Specifically, univariate general linear models (GLMs) tested the effects of separate *APOE* and *TOMM40* genotypes upon left/right hippocampal volumes. Raw *P*-values < 0.05 were considered significant. 

It is important to correct (commonly and herein ‘normalize’) left and right hippocampal volumes relative to an individual’s head or brain size. To this end, left/right hippocampal volumes were analysed in three separate ways, controlling for age and gender throughout. First, left/right hippocampal volumes were examined raw. Second, hippocampal volumes were analysed with intracranial volume as an additional model covariate, reflecting maximum lifetime brain volume as a general proxy for head size. Finally, hippocampal volumes were analysed with current total brain tissue volume as an additional model covariate, to ensure that any significant genetic associations with hippocampal volume were not reflective of more general brain atrophy [52]. 

Regression-based normalization more comprehensively eliminates variance in hippocampal volume that is associated with intracranial or current brain tissue volumes, compared with proportional percentile- or ratio-based corrections [52–54]. Rather than creating new variables reflecting ‘left/right hippocampal volumes residualized for intracranial/total brain tissue volumes’, where appropriate we included intracranial/total brain tissue volumes as model covariates in addition to gender and age. This is preferable because it also adjusts for any effects that gender and age may have on associations between hippocampal and intracranial/total brain tissue volumes [55]. 

### *APOE* analysis

The *APOE* ε haplotype is composed of any two of the ε2 (protective), ε3 (neutral) and ε4 (risk) alleles. The first analytic step tested the effects of *APOE* ε4 allele presence vs. absence, i.e. pooled ε2/ ε4, ε3/ε4 & ε4/ ε4 genotypes vs. pooled ε2/ ε2, ε2/ε3 & ε3/ ε3 (‘Step 1’). In the next step, genotypes which may be protective for neurodegenerative pathology were compared with the neutral genotype; i.e.; pooled ε2/ε3 & ε2/ε2 against ε3/ε3 (‘Step 2’) [9,56]. 

### *TOMM40* analysis

Length of the variable-length poly-T repeat rs10524523 (‘523’) was split into three categories: ‘S’ (<20 T residues), ‘L’ (20-29) and ‘VL’ (≥ 30) [21]. In the first analytic step, in the whole sample, a GLM tested for a significant effect of *TOMM40* 523 genotype (i.e. S/S; S/L; L/L; L/VL; VL/VL; ‘Step 1’). To investigate the effects of *TOMM40* 523 repeat length independent of *APOE* ε genotype, analysis focussed separately on two different *APOE* ε subgroups; firstly participants with the ε3/ε4 genotype (‘Step 2’). Devi et al.[57] reported that in a sample of adults diagnosed with AD, this genotype had the highest accumulation of amyloid precursor protein (APP) in mitochondria (versus ε3/ε3 and ε4/ε4 genotypes; assessed by immunoblot analysis). This accumulation correlated significantly with two indicators of mitochondrial dysfunction, namely cytochrome C oxidase activity and reactive oxygen species hydrogen peroxide (‘H_2_O_2_’). Presence of the S allele may play a role in a possible interaction between APP accumulation/mitochondrial translocase processes [23]. Finally, analysis focussed on participants with the neutral *APOE* genotype (ε3/ε3) (‘Step 3’), because this eliminates variance associated with protective and risk *APOE* alleles [3]. 

In large sample of Caucasians, linkage between the *APOE* ε genotype and *TOMM40* 523 length (i.e. ε4 links primarily to ‘L’, ε3 primarily to ‘S’ or ‘VL’) is such that in the *APOE* ε3/ε3 genotype, relatively few L carriers would be predicted while in the ε3/ε4 genotype typically one L allele would be predicted in addition to either an S or VL allele [50]. Slight errors in poly-T repeat length measurement may occur through PCR ‘slippage’ and this may result in repeat lengths that are close to the L/VL boundary being incorrectly classified [50]. To attempt to control for this, in Steps 2 and 3, the L and VL alleles were pooled into an ‘L*’ group; participants with the S/S genotype were compared with those carrying only one S allele (pooled S/L and S/VL; hereinafter S/L*), and also against participants carrying no S alleles (pooled L/L, L/VL and VL/VL; hereinafter L*/L*) [58]. A GLM therefore tested for effects of S-allele dosage (S/S; S/L*; L*/L*) on hippocampal volume variables in Steps 2 and 3 (*APOE* ε3/ε4 and ε3/ε3 subgroups respectively).

## Results

### Descriptive statistics

Of the 1091 total LBC1936 participants, 866 attended Waves 1 and 2, and 700 underwent neuroimaging. Individuals with MRI data were excluded if they had MMSE scores below 24 (n = 5) or not completed at Wave 2 (n = 1). No participants reported dementia. Overall, this left 694 participants of which 655 had successfully segmented left and right hippocampal volumes. Demographic and clinical statistics for the sample are shown in Table 2. Of these, 624 and 636 participants had successful genotyping for *APOE* ε and *TOMM40* 523, respectively. 

**Table 2:** Basic demographic and clinical data for the current sample.

	N
Current sample N	655
Gender: N that are female (% of total)	312 (47.6)
Age in years at clinic visit near to time of MRI (SD)	72.7 (0.7)
MMSE score (SD)	28.9 (1.3)
History of hypertension: N (% of total)	324 (49.5)
History of diabetes: N (% of total)	67 (10.2)
History of high cholesterol: N (% of total)	275 (42.0)
History of other cardiovascular disease: N (% of total)	179 (27.3)
History of stroke: N (% of total)	44 (6.7)

Note. SD = standard deviation, MMSE = Mini-Mental State Exam (maximum score of 30). These data are based on the sample of participants with successfully segmented hippocampal volumes, once those participants with reported dementia, or with MMSE scores ≤24 (or missing) around the time of MRI, were removed.

*APOE* had allele frequencies of ε2 = 7.4%, ε3 = 76.9% and ε4 = 15.7%, with genotype frequencies of: ε2/ε2 = 2 (0.3%), ε2/ε3 = 83 (12.0%), ε2/ε4 = 15 (2.2%), ε3/ε3 = 401 (58.2%), ε3/ε4 = 175 (25.4%) and ε4/ε4 = 13 (1.9%). *TOMM40* 523 had allele frequencies of S = 41.0%, L = 15.4% and VL = 43.6%, with genotype frequencies of S/S = 106 (15.2%), S/L = 103 (14.7%), S/VL = 259 (37.1%), L/L = 16 (2.3%), L/VL = 80 (11.4%) and VL/VL = 135 (19.3%). Exact tests confirmed that *APOE* ε and *TOMM40* 523 genotypes were in Hardy-Weinberg equilibrium (*P* values = 0.449 and 0.111 respectively). Descriptive statistics for brain imaging variables are shown in Table 3, and intercorrelations between intracranial, total brain tissue and hippocampal volumes in mm^3^ are presented in Table 4, showing medium to strong statistically significant intercorrelations. Reported volumes are similar to those in independent samples [52]. 

**Table 3:** Descriptive statistics for raw hippocampal, intracranial and total brain tissue volumes, grouped by genotype.

Genotype (mean; standard error)	Left raw hippocampal volume	Right raw hippocampal volume	Intracranial volume	Total brain tissue volume	Left hippocampal volume (ICV-corrected)	Right hippocampal volume (ICV-corrected)	Left hippocampal volume (TBV-corrected)	Right hippocampal volume (TBV-corrected)
*APOE* ε								
ɛ4 absent	3094.41 (20.36)	3335.06 (19.98)	1,442,258.32 (4985.87)	1,119,764.90 (4316.47)	3104.24 (19.46)	3344.71 (19.10)	3100.36 (18.95)	3341.09 (18.48)
ɛ4 present	3084.16 (31.25)	3317.20 (30.67)	1,468,448.23 (7694.48)	1,130,853.17 (6671.72)	3061.01 (29.96)	3294.47 (29.40)	3070.30 (29.19)	3302.68 (28.46)
ɛ2 present	3038.94 (46.42)	3315.15 (45.79)	1,449,976.37 (11,760.64)	1,127,564.58 (10,219.88)	3031.03 (43.36)	3308.35 (43.53)	3024.76 (42.08)	3301.68 (41.85)
ɛ3/ɛ3	3103.94 (21.33)	3337.77 (21.04)	1,440,660.88 (5400.47)	1,127,674.58 (4663.18)	3105.62 (19.92)	3339.22 (20.00)	3106.96 (19.33)	3340.64 (19.22)
*TOMM40* ‘*523*’								
Short/Short	3135.63 (44.01)	3360.83 (43.15)	1,442,116.02 (10,827.43)	1,126,441.85 (9377.16)	3144.34 (41.91)	3369.50 (41.02)	3133.37 (40.89)	3358.46 (39.79)
Short/Long	3174.39 (43.76)	3401.25 (42.90)	1,473,374.04 (10,880.91)	1,137,288.23 (9472.76)	3146.15 (41.81)	3373.14 (40.92)	3154.67 (40.93)	3380.33 (39.83)
Short/Very-Long	3076.52 (27.96)	3331.20 (27.41)	1,436,902.51 (6871.98)	1,116,375.69 (5951.29)	3093.33 (26.69)	3347.93 (26.13)	3087.99 (25.99)	3342.88 (25.30)
Long/Long	3104.79 (110.00)	3331.20 (27.41)	1,487,577.11 (27,496.05)	1,145,780.72 (23,812.37)	3059.36 (104.87)	3356.94 (102.65)	3066.82 (102.25)	3363.22 (99.52)
Long/Very-Long	3028.12 (49.65)	3402.16 (107.85)	1,463,850.24 (12,244.22)	1,127,040.17 (10,603.84)	3009.19 (47.32)	3243.97 (46.32)	3017.10 (46.13)	3251.47 (44.90)
Very-Long/Very-Long	3057.69 (37.24)	3301.85 (36.51)	1,450,239.49 (9307.98)	1,119,956.79 (8060.97)	3057.88 (35.45)	3302.04 (34.70)	3064.65 (34.60)	3308.92 (33.67)

Note. All volumes are in mm^3^, estimated marginal means adjusted for age and gender. ICV-corrected = additionally adjusted for intracranial volume, TBV-corrected = additionally adjusted for total brain tissue volume.

**Table 4:** Inter-correlations between hippocampal, intracranial and total brain tissue volumes.

	Left hippocampal volume	Right hippocampal volume	Intracranial volume	Total brain tissue volume
Left hippocampal volume	-	0.78	0.40	0.47
Right hippocampal volume	0.78	-	0.45	0.50
Intracranial volume	0.40	0.45	-	0.87
Current brain tissue volume	0.47	0.50	0.87	-

Note. Unadjusted Pearson bivariate correlations. All volumes are raw in mm^3^, and figures reflect ‘*r*’ correlations. All associations were significant at P <0.001.

### *APOE* ε, *TOMM40* 523 poly-T repeat and hippocampal volumes

There was no effect of the *APOE* ε4 present vs. absent comparison (Step 1), nor pooled ε2/ε3; ε2/ε2 genotype (vs. ε3/ε3; Step 2) on hippocampal volumes analysed raw, or normalized for either intracranial or total brain tissue volumes (all P > 0.05; see Table 5). 

**Table 5:** Apolipoprotein-e (*APOE*) ε genotype and hippocampal volumes.

Volume in mm^3^	ɛ4 allele presence vs. absence			ɛ2/ɛ3 & ɛ2/ɛ2 vs. ɛ3/ɛ3		
	d.f. & F statistics	*P*	Partial η^2^	d.f. & F statistics	*P*	Partial η^2^
Left raw hippocampal volume	1, 620 = 0.08	0.784	0.000	1, 434 = 1.61	0.205	0.004
Right raw hippocampal volume	1, 620 = 0.24	0.626	0.000	1, 434 = 0.20	0.654	0.000
Left hippocampal volume (ICV-corrected)	1, 619 = 1.45	0.228	0.002	1, 433 = 2.43	0.120	0.006
Right hippocampal volume (ICV-corrected)	1, 619 = 2.04	0.154	0.003	1, 433 = 0.41	0.521	0.001
Left hippocampal volume (TBV-corrected)	1, 618 = 0.75	0.388	0.001	1, 433 = 3.12	0.077	0.007
Right hippocampal volume (TBV-corrected)	1, 618 = 1.28	0.258	0.002	1, 433 = 0.71	0.399	0.002

Note. Age at time of testing and gender statistically controlled. ICV-corrected = intracranial volume included as a covariate, TBV-corrected = total brain tissue volume included as a covariate.

No effects of *TOMM40* 523 poly-T repeat genotype were found in the whole sample (Step 1), nor in subgroup analyses of *APOE* ε3/ε4 (Step 2) or ε3/ε3 genotypes specifically (Step 3) for any of raw or normalized hippocampal volumes (all *P* > 0.05; see Table 6).

**Table 6:** Translocase of outer membrane 40 (*TOMM40*) ‘*523*’ poly-T repeat genotype and hippocampal volumes.

Volume in mm^3^	Whole sample			ɛ3/ɛ4 genotype subgroup			ɛ3/ɛ3 genotype subgroup		
	d.f. & F statistics	*P*	Partial η^2^	d.f. & F statistics	*P*	Partial η^2^	d.f. & F statistics	*P*	Partial η^2^
Left raw hippocampal volume	5, 628 = 1.47	0.198	0.012	1, 156 = 1.42	0.235	0.009	2, 349 = 1.32	0.268	0.008
Right raw hippocampal volume	5, 628 = 1.23	0.292	0.010	1, 156 = 0.61	0.435	0.004	2, 349 = 1.57	0.210	0.009
Left hippocampal volume (ICV-corrected)	5, 627 = 1.47	0.198	0.012	1, 155 = 1.22	0.271	0.008	2, 348 = 1.15	0.316	0.007
Right hippocampal volume (ICV-corrected)	5, 627 = 1.33	0.250	0.010	1, 155 = 0.429	0.513	0.003	2, 348 = 1.70	0.184	0.010
Left hippocampal volume (TBV-corrected)	5, 626 = 1.34	0.244	0.011	1, 154 = 1.55	0.216	0.010	2, 348 = 0.79	0.455	0.005
Right hippocampal volume (TBV-corrected)	5, 626 = 1.18	0.319	0.009	1, 154 = 0.67	0.415	0.004	2, 348 = 1.30	0.274	0.007

Note. Age at time of testing and gender statistically controlled. ICV-corrected = intracranial volume included as a covariate, TBV-corrected = total brain tissue volume included as a covariate.

### Additional comparative analysis

To permit comparison with other reports that use alternative methods of normalization for head size – namely, reporting hippocampal volumes as an absolute proportion of total intracranial volume – the above main results were re-run using the formula “Left-or-right hippocampal volume in mm^3^ / intracranial volume in mm^3^ x 1000” [59]. Re-analysis showed that the main results were for the most part unchanged, i.e. not significant at *P* < 0.05. One exception was that for the *APOE* ε4 present vs. absent comparison, there was a significant deleterious effect of the ε4 allele for the right hippocampus (F [1, 620] = 4.54, *P* = 0.034, η^2^ = 0.007). 

To check the extent to which left and right hippocampal ‘ratios’ were independent of intracranial volume, unadjusted bivariate correlations were run. Intracranial volume correlated significantly with left (*r* = -0.26) and right (*r* = -0.28) hippocampal volumes expressed as a proportion of intracranial volume (both *P* < 0.001), indicating a lack of true independence. 

## Discussion

### Overview

The current study investigated the effects of the *APOE* ε and *TOMM40* rs10524523 (‘*523*)’ poly-T repeat gene loci on hippocampal volumes - raw and also corrected (commonly and herein referred to as ‘normalized’) separately for intracranial and total brain tissue volumes - in the LBC1936. This normalization was implemented because we aimed to test for genetic influence on the hippocampus independent of any possible effects on intracranial or total brain tissue volumes. We report no significant effects of genetic variation at either the *APOE* ε or *TOMM40* 523 loci, on left or right hippocampal volumes, when analysed either raw or normalized by intracranial or total brain tissue volumes. 

Hippocampal volumes and *APOE* have been investigated in a number of previous reports, the majority of which were relatively small (N < 135). *TOMM40* 523 and hippocampal volumes have been investigated by one previous study [43], however that report examined specifically participants with the *APOE* ε3/ε3 genotype (N = 117), and found no significant associations. The current study tested the *TOMM40 523* locus in a larger sample of older adults and included all *APOE* ε genotypes (n = 623), ε3/ε4 carriers only (n = 160), and ε3/ε3 carriers only (n = 376), and found no effect of poly-T repeat length genotype.

### Interpretation: *APOE* ε

Previous large studies have reported significant effects of *APOE* ε genotype on hippocampal volumes in healthy older adults [37,38]. In a similarly large sample of older adults, we did not replicate these, and instead echo other large reports which show no association with genetic variation at the *APOE* locus [39–41]. There is no evidence that large positive studies by LeMaitre et al. [37] (mean age = 69.2; mean MMSE = 27.3) or Den Heijer et al.[38] (mean age = 72.0 years; mean MMSE score = 27.4) were composed of markedly more cognitively impaired or younger/older subjects than those reported here (mean age = 72.7, mean MMSE = 28.8). The discrepancy may reflect type 1 error in previous reports. Part of the discrepancy may also relate to normalization technique. Previous studies vary in how they normalize left and right hippocampal volumes - namely they use either ‘ratio’ (e.g. Lemaitre et al.[37]) or ‘covariance’ techniques (e.g. Den Heijer et al. [38]). Above in our main results we report on hippocampal volumes covaried separately for intracranial and brain tissue volumes as we believe this to be the most appropriate correction (see below [52–54]).

To permit comparison with other reports, the main results were additionally re-analysed with hippocampal volumes normalized as a ratio of intracranial volume. The results were unchanged except for a significant deleterious effect of *APOE* ε4 allele presence vs. absence. However, we judge that statistically controlling for intracranial volume is the more appropriate normalization technique for the following reasons:

1 Ratio measures do not completely eliminate association between head size and hippocampal volume [52–54]. Further analysis showed that hippocampal volumes expressed as a ratio of intracranial volume correlated significantly with intracranial volume itself. Ratios therefore do not allow for an assessment of *APOE*, *TOMM40* and hippocampal volume completely independent of head size, which was the main aim of the current study.
2 Any disparity between genotype groups as assessed by ratios may reflect differences in any of the numerator (hippocampus), the denominator (intracranial volume) or their interaction, reduced to one variable. This is less informative than regression techniques which take into account the strength of association between the specific brain structure and the larger denominator [52,54].

Critically reviewing previous reports further, we are aware of no study that corrects significant *APOE*-hippocampal volume associations for current total brain tissue volume. MacLullich et al.[60] reported in a sample of older adults (N = 97, age range = 65-70 years), that left/right hippocampal, frontal lobe, temporal lobe and intracranial volumes strongly and positively intercorrelated with one another (*r* range = 0.29 to 0.83, all P < 0.005). Data reduction with principal component analysis showed that these loaded strongly and significantly onto a ‘general brain size’ factor (range of loadings = 0.64 to 0.73, *P* < 0.05). Intracranial volume is relatively constant throughout the lifespan, while actual brain tissue volume is susceptible to age-related change[61]. Given that we are aware of no previous study that reports significant *APOE*-hippocampal volume associations in older adults that also controls for current total brain tissue volume, we cannot exclude the possibility that those associations may be secondary to more generalized brain atrophy[38][60,62][63]. (Note however that Den Heijer et al. [38] reported no significant ε4 effect on semi-qualitative observer-rated cortical atrophy; ranged 0-3 at various locations). It is also unclear whether previous large significant positive studies have assumed normal distributions for normalized hippocampal volumes in older-age samples, as this may introduce errors in analysis and increase the risk of spurious results. 

### Interpretation: *TOMM40* ‘523’ poly-T repeat

No effects of *TOMM40* 523 were found, and this could be cautiously interpreted in terms of different explanations, given the absence of further relevant data. Specifically:

1 The *TOMM40 523* repeat does not significantly affect mitochondrial function [20,22,64].
2 *TOMM40* 523 locus does affect mitochondrial function, but not to an extent that affects hippocampal volume (the outcome variable) in this sample: the mitochondrial cascade hypothesis describes a ‘threshold’ beyond which mitochondrial mutations are not adequately compensated for and significant histology resembling AD emerges [25,29]. Perhaps hippocampal volume assessed by MRI is unaffected before this threshold [19].
3 The effect of *TOMM40* 523 length is moderated by or interacts with additional genetic variants for example loci in LD with *APOE* or *TOMM40* such as *APOC1* [20]. It may also be possible to investigate the possibility of moderation of *TOMM40*/*APOE* effects by other genetic factors; however, despite the relatively large sample size (by brain imaging standards), this would be statistically challenging.

### Future research

Specific subregions of the hippocampal formation may be more vulnerable to brain ageing or incipient AD pathology, and therefore more sensitive to variations at specific relevant genetic loci. Devanand et al.[65] reported that subregions of the hippocampus differentially predicted longitudinal diagnosis of clinical AD over three years, in a sample of individuals with amnestic mild cognitive impairment (N = 130, of which 31 converted to AD; baseline MMSE scores all >22). Controlling for age, gender, years of education, and intracranial volume, cox regression analyses showed that volumes in the cornu ammonis 1 (left hazard ratio = 0.22, P = 0.054, right hazard ratio = 0.23, P = 0.06) and subiculum subregions (left hazard ratio = 0.22, P = 0.054, right hazard ratio = 0.22, p = 0.03) were more predictive of longitudinal AD diagnosis compared with entorhinal cortical volume (right hazard ratio = 0.05, P = 0.06, left hazard ratio = 0.02, P = 0.26). Future studies of hippocampal volume in the LBC1936 may therefore consider more fine-grained analysis of the hippocampal formation as genetic variation at the *APOE* or *TOMM40* loci may affect specific subregions first. Functional brain MRI may also be a more sensitive marker of hippocampal dysfunction, compared with left/right volumes as assessed here by structural MRI.

 It is possible that the current sample of non-demented, generally healthy older adults (aged around 73 years) have not undergone sufficient volumetric hippocampal or brain atrophy to show significant differentiation according to genotype. The sample examined here is undergoing repeat structural brain MRI, around the age of 76. Significant associations with *APOE* or *TOMM40* genotypes may become apparent at this older age, after more age-related atrophy. 

## Summary

This study examined the independent effects of variation at the *APOE* ɛ and the *TOMM40 523* poly-T repeat gene loci upon hippocampal volume assessed raw and also normalized separately for intracranial and total brain tissue volumes. Previous large studies have occasionally reported significant associations between the *APOE* ε4 allele and lower hippocampal volumes. The current study does not replicate those significant reports in a community dwelling sample of older adults of homogenous ages. Previous significant findings may reflect type 1 error, or partly reflect discrepancies in how the hippocampus has been normalized relative to head size in different studies. We can see no obvious evidence that previously examined healthy older samples differed markedly from the current sample, either in terms of age or prevalence of cognitive decline (based on average MMSE scores). Studies that show significant genetic associations with the hippocampus should run further confirmatory analysis to investigate whether associations are independent of general volumetric brain atrophy. We also found no significant effects of *TOMM40* 523 poly-T repeat length. Future studies may investigate specific subregions of the hippocampal formation; there may be effects of the *APOE* or *TOMM40 523* genetic loci that are not manifest in overall hippocampal volume. 
